# Intravascular large B-cell lymphoma associated with silicone breast implant, HLA-DRB1*11:01, and HLA-DQB1*03:01 manifesting as macrophage activation syndrome and with severe neurological symptoms: a case report

**DOI:** 10.1186/s13256-016-0993-5

**Published:** 2016-09-15

**Authors:** Oswald Moling, Andrea Piccin, Martina Tauber, Peter Marinello, Mariagrazia Canova, Marco Casini, Giovanni Negri, Bernd Raffeiner, Raffaella Binazzi, Latha Gandini, Cinzia Vecchiato, Giovanni Rimenti, Atto Billio

**Affiliations:** 1 Division of Infectious Diseases, Ospedale Generale, 39100 Bolzano, Italy; 2 Department of Hematology, Ospedale Generale, 39100 Bolzano, Italy; 3 Department of Pathology, Ospedale Generale, 39100 Bolzano, Italy; 4 Department of General Surgery, Ospedale Generale, 39100 Bolzano, Italy; 5 Rheumatology Unit, Department of Medicine, Ospedale Generale, 39100 Bolzano, Italy; 6 Laboratory of Immunogenetics, Transfusion Medicine Service, Ospedale Generale, 39100 Bolzano, Italy

**Keywords:** Silicone breast implant, Siliconosis, Autoimmune inflammatory syndrome induced by adjuvants (ASIA), Macrophage activation syndrome (MAS), Hemophagocytic lymphohistiocytosis (HLH), Intravascular large B-cell lymphoma (IVLBCL)

## Abstract

**Background:**

Silicone implants have been successfully used for breast augmentation and reconstruction in millions of women worldwide. The reaction to the silicone implant is highly variable; it can lead to local inflammatory symptoms, and sometimes to systemic symptoms and disease. Over 80 cases of anaplastic lymphoma kinase-negative anaplastic large cell lymphoma have been reported in patients with silicone breast implants and have been accepted as a new clinical entity. To the best of our knowledge, an intravascular large B-cell lymphoma associated with a silicone breast implant has not been reported previously.

**Case presentation:**

A 48-year-old Caucasian woman who presented with high fever was found to have splenomegaly on physical examination. A laboratory diagnosis revealed pancytopenia, hypertriglyceridemia, and hyperferritinemia. She developed signs of altered sensorium, hemiparesis, aphasia, and cauda equina syndrome. On further evaluation, she fulfilled the necessary five out of eight criteria for diagnosis of macrophage activation syndrome/hemophagocytic lymphohistiocytosis. Dexamethasone administration was followed by prompt improvement; however, 3 days later she again manifested high fever, which persisted despite administration of immunoglobulin and cyclosporine A. Her silicone breast implant was considered a possible contributor to her macrophage activation syndrome and was therefore removed. A histological examination of the capsule tissue showed an extensive lymphohistiocytic/giant cell foreign body reaction suggestive of autoimmune/inflammatory syndrome induced by adjuvants. However, the histological examination unexpectedly also revealed an intravascular large B-cell lymphoma.

**Conclusions:**

The genetic background of our patient with silicone breast implants might have predisposed her to three rare and difficult to diagnose syndromes/diseases: macrophage activation syndrome/hemophagocytic lymphohistiocytosis, autoimmune/inflammatory syndrome induced by adjuvants, and intravascular large B-cell lymphoma. The simultaneous manifestation of all three syndromes suggests causal interrelationships. Human leukocyte antigen testing in all women who undergo silicon breast implantation could in the future enable us to better evaluate the risk of potential side effects.

**Electronic supplementary material:**

The online version of this article (doi:10.1186/s13256-016-0993-5) contains supplementary material, which is available to authorized users.

## Background

Hemophagocytic lymphohistiocytosis (HLH), also referred to as hemophagocytic syndrome, is a life-threatening hyperinflammatory syndrome that can occur in many underlying conditions: homozygous mutations affecting the cytotoxic function of natural killer (NK) cells and cytotoxic T cells (CTLs), infections, autoimmune diseases, malignancies, metabolic diseases, acquired immunodeficiency such as AIDS, iatrogenic immunosuppression, and solid organ or stem cell transplantation [1–3]. It is caused by an exaggerated or persistent immune stimulation and/or the failure to downregulate or to end an immune response [1]. It can be seen as a cytokine storm disorder, representing a variety of inflammatory etiologies with the final common result of overwhelming inflammation, hemodynamic instability, multiple organ failure, and potentially death [4]. HLH can be differentiated into primary (also called familial, hereditary, or genetic) and secondary (also called sporadic, reactive, or acquired) HLH [1, 2]. For the diagnostic criteria proposed by the Histiocyte Society in 2004, see Table 1 [5]. Macrophage activation syndrome (MAS) is an acquired form of HLH that occurs in autoimmune diseases, and it is mostly reported in patients with systemic juvenile idiopathic arthritis (sJIA), and less commonly in those with systemic lupus erythematosus (SLE), adult-onset Still’s disease (AOSD), rheumatoid arthritis (RA), spondyloarthropathy, and vasculitis [1, 6, 7]. There are no universally accepted criteria for MAS, thus many clinicians simply refer to the HLH criteria. Diagnosis of MAS can be difficult and it is hard to distinguish from flares of the underlying disease, and from sepsis-like syndromes, although it may also be associated with sepsis, especially in cases of intraphagocytic infections [6]. However, preliminary MAS diagnostic guidelines have shown a better capacity to identify MAS in sJIA and to differentiate it from systemic infections compared to the 2004-HLH diagnostic criteria [8].

**Table 1:** Diagnostic criteria for hemophagocytic lymphohistiocytosis

Diagnosis of HLH is based on the presence of 5 or more of the following: • Fever • Splenomegaly • Cytopenias (affecting 2 of 3 lineages in the peripheral blood) Hemoglobin <90 g/L Platelets <100 × 10^9^/L Neutrophils <1.0 × 10^9^/L • Hypertriglyceridemia and/or hypofibrinogenemia Fasting triglycerides >3.0 mmol/L (>263 mg/dL) Fibrinogen <1.5 g/L • Hemophagocytosis in bone marrow, spleen, or lymph nodes • Low or absent NK-cell activity • Ferritin >500 μg/L • Soluble IL-2 receptor >2400 U/mL	

Adapted from the Histiocyte Society HLH-2004 protocol [5]

*HLH* hemophagocytic lymphohistiocytosis, *IL* interleukin, *NK* natural killer

Autoimmune/inflammatory syndrome induced by adjuvants (ASIA) or Shoenfeld’s syndrome is a recently coined term for a spectrum of syndromes that includes macrophage myofascitis syndrome, Gulf war syndrome, post-vaccination phenomena, and siliconosis [9–11]. For the proposed criteria for the diagnosis of ASIA, see Table 2 [9, 12]. Silicone implants have been successfully used for breast augmentation and reconstruction in millions of women worldwide [13]. However, case reports and case series on side effects associated with silicone breast implants (SBIs) were published soon after their first application [14–24]. Anecdotal cases of breast cancer following SBI have been reported, too [25–27], however, epidemiological studies have not shown any evidence correlating malignancies with SBIs [28, 29]. Nevertheless, case reports of carcinomas and lymphomas continue to be published and no definitive consensus opinion has been obtained [30–33]. Meanwhile, over 80 cases of anaplastic lymphoma kinase (ALK)-negative anaplastic large cell lymphoma have been reported in patients with SBIs and it has been accepted as a new clinical entity [33]. Among the autoimmune connective tissue diseases diagnosed in patients with SBI, scleroderma has been reported most often; other diagnoses included SLE, RA, Sjögren’s syndrome, and mixed connective tissue diseases [16–18, 20, 21, 24]. Again, epidemiological studies and meta-analysis have rejected the presumed relationship between SBIs and autoimmune connective tissue diseases [13, 34] but, in seeming contradiction, case reports about autoimmune connective tissue diseases continue to be published [10, 23, 24]. However, a relationship between SBIs and a particular constellation of symptoms that did not fulfill diagnostic criteria for any recognized autoimmune connective tissue diseases has been documented in several studies [10, 13, 19, 22, 23, 34, 35]. Reported symptoms of these non-defined autoimmune phenomena are fatigue, muscular weakness, morning stiffness, arthralgia, myalgia, dry eyes, dry mouth, frequent sore throats, night sweats, rash, Raynaud’s phenomena, alopecia, adenopathy, poor sleep, headache, memory loss, and sensory loss [10, 13, 19, 20, 23, 34, 35]. In addition, the localized and very variable foreign body inflammatory reaction to the SBI can lead to the formation of an excessive fibrous capsule and capsular contracture, which occur in 2–50 % of individuals [36].

**Table 2:** Suggested criteria for the diagnosis of autoimmune/inflammatory syndrome induced by adjuvants [9]

Major criteria • Exposure to an external stimulus (infection, vaccine, silicone, adjuvant) prior to clinical manifestations • The appearance of ‘typical’ clinical manifestations: Myalgia, myositis, or muscle weakness Arthralgia and/or arthritis Chronic fatigue, un-refreshing sleep, or sleep disturbances Neurological manifestations (especially associated with demyelination) Cognitive impairment, memory loss Pyrexia, dry mouth • Removal of inciting agent induces improvement • Typical biopsy of involved organs Minor criteria • The appearance of autoantibodies or antibodies directed at the suspected adjuvant • Other clinical manifestations (e.g., irritable bowel syndrome) • Specific HLA (e.g., HLA DRB1…, HLA DQB1…) • Evolvement of an autoimmune disease (e.g., multiple sclerosis, systemic sclerosis)	

*HLA* human leukocyte antigen

An evaluation of these criteria in 93 cases of ASIA following hepatitis B vaccine revealed that fulfillment of either two major or of one major and two minor criteria is required to diagnose ASIA [12]

Intravascular large B-cell lymphoma (IVLBCL) is a rare subtype of diffuse large B-cell lymphoma [37–39]. It is characterized by proliferation and aggregation of clonal lymphocytes within the lumina of capillaries, arterioles, and venules. The surprising degree of sparing of the surrounding tissue and the absence of lymphoma cells in the lymph nodes is a hallmark of the disease [37–40]. This lymphoma is extremely heterogeneous in its clinical presentation and has been described in the small vessels of nearly every organ, leading to ischemia, organ dysfunction, and organ failure [37–40]. Therefore, it has also been called the oncologist’s “great imitator.” The majority of cases can be grouped into few discrete presentations: central nervous system involvement or cutaneous involvement (also called Western variant), fever of unknown origin, hemophagocytic syndrome/HLH, and multi-organ failure (also called Asian variant). An exhaustive search for an infectious etiology often contributes to the delay in diagnosis [39]. Up to two thirds of patients have neurologic manifestations [41]. The following syndromes have been found to be associated with IVLBCL: cerebrovascular events, encephalopathy, myelopathy and/or cauda equina syndrome, and peripheral or cranial neuropathies [41–44]. Beyond these presentations, there are single case reports of IVLBCL presenting primarily in other organ systems, for example, as interstitial lung disease, adrenal failure, pulmonary hypertension, nephrotic syndrome, myocardial infarction, and symmetric polyarthritis. Given the rarity of IVLBCL, its multiplicity of presentations, and its absence in lymph nodes (where lymphomas are usually localized), it is an extremely difficult diagnosis to make ante mortem [39]. IVLBCL can be fatal if diagnosis and treatment are delayed. It has emerged that ^18^Fluorodeoxy-glucose positron emission tomography/computed tomography (^18^FDG-PET/CT) may be useful to allow suspicion of a diagnosis of IVLBCL, to detect unexpected locations, to guide diagnostic biopsy, and to assess the response to treatment [45]. A random skin biopsy from normal-appearing skin has been shown to be highly sensitive in the diagnosis of ILVBCL and should be performed irrespective of the presence or absence of skin lesions in patients in whom IVLBCL is suspected [46]. Random transbronchial lung biopsies can be diagnostic in patients affected by IVLBCL either with negative ^18^FDG-PET/CT imaging or with no abnormal chest computed tomography [47, 48].

## Case presentation

A 48-year-old Caucasian woman was referred to our hospital to exclude an unrecognized infection as the cause of three episodes of fever associated with splenomegaly. Her past medical history was unremarkable up to 4 years previously, when by routine mammography and subsequent biopsy an intraductal carcinoma was diagnosed. She underwent a skin-sparing mastectomy of her right breast combined with reconstruction and implantation of a silicone prosthesis (Polytech 445 g). No adjuvant chemotherapy or radiotherapy was provided or indicated. Three years later she underwent a reduction operation of her left breast combined with lipofilling of both breasts. She had experienced fever 8 and 5 months prior to hospitalization that lasted for about 3 weeks. She was treated at home with levofloxacin because of a known allergy to penicillin.

When fever appeared again together with sore throat, fatigue, sweating, cough (she smokes about 10 cigarettes daily), and an involuntary weight loss of 10 kg in the last year, she was hospitalized for 2 weeks in a peripheral hospital. Abnormal results from laboratory investigations included: hemoglobin 10.4 g/dL, leukocytes 3.6 × 10^9^/L, serum total protein 5.3 g/dL, lactate dehydrogenase (LDH) 638 U/L, and ferritin 398 ng/mL. An abdominal ultrasound examination demonstrated her spleen was enlarged, with a diameter of 15 cm. X-ray examination of her chest showed bilateral apical pleural scarring, which was confirmed by computed tomography (CT). CT also revealed subpleural densities of uncertain significance, an enlarged liver and spleen, ovarian cysts, and a 2.3 cm large layer of fluid in the Douglas space. There was no evidence of enlarged mediastinal, abdominal, or retroperitoneal lymph nodes. A histologic examination of a bone marrow biopsy specimen was of no diagnostic help. ^18^FDG-PET/CT evidenced hypermetabolism of both adrenal glands. She was given azithromycin and her fever subsided but profound weakness remained. Extensive laboratory examinations did not indicate any infectious, autoimmune, or malignant diseases. She was referred to our Division of Infectious Diseases for a search for an unrecognized infectious disease.

On physical examination our patient was pale and weak. Her body temperature was 37.3 °C, blood pressure 110/90 mmHg, heart frequency 92 beats/minute, arterial blood oxygen saturation 98 %, body weight 59 kg, and high 168 cm. She complained of memory deficits and an unexplainable unstable gait. There were no signs of meningeal irritation. Since her breast operation 4 years previously she had needed sleeping tablets. During her first 6 days in hospital without any treatment her body temperature rose progressively from 37.3 to 39 °C. Because of the pleural scars and subpleural densities seen in the CT images, and the hypermetabolic adrenal glands shown by ^18^FDG-PET/CT [adrenocorticotropic hormone (ACTH) and cortisol concentrations were within the normal range], a tuberculosis (TB) infection was considered. Results from a repeated search by microscopy, culture and PCR analysis of *Mycobacterium tuberculosis* in her sputum and bronchial aspirate were unremarkable. A Mantoux skin test and QuantiFERON-TB assay were negative too. *Leishmania* infection was excluded by serology and microscopy of her bone marrow aspirate.

**Fig. 1:**
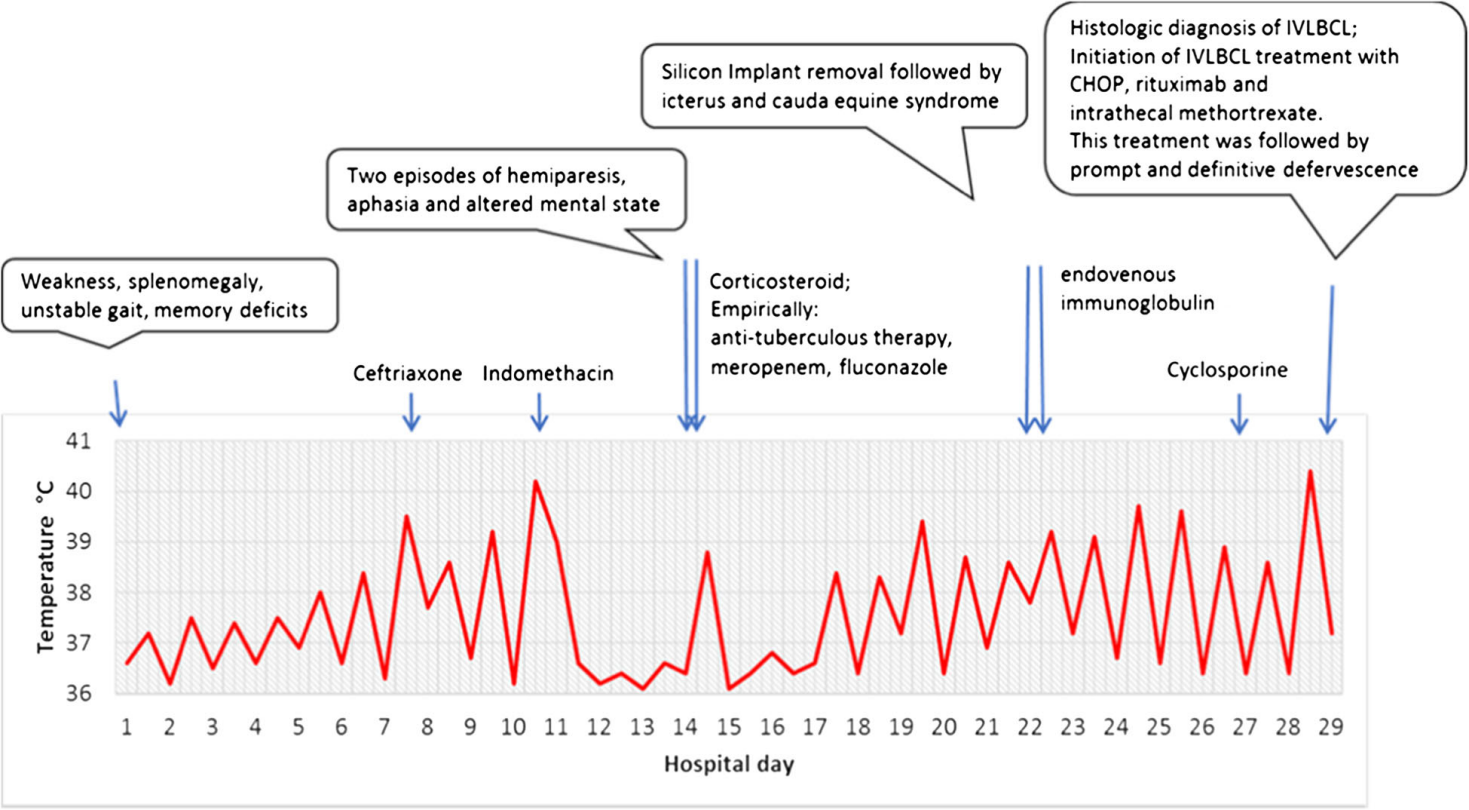
Chronological sequence of the main clinical features until the final diagnosis of intravascular large B-cell lymphoma (*ILBCL*) was made. *CHOP* cyclophosphamide, hydroxydaunorubicin (doxorubicin), vincristine, prednisone

Despite repeated negative blood, urine, and stool cultures for pathogenic bacteria, among the many laboratory examinations, titers of 1:160 anti-Salmonella O and 1:2560 anti-Salmonella H antibodies were detected by Widal Wright’s reaction (Table 3). Thus empiric antibiotic therapy with ceftriaxone 2 g daily was started. There was no response to treatment and her high fever persisted. Therefore, indomethacin 50 mg three times daily was administered. This was followed by prompt defervescence; however, it lasted only for 3 days (Fig. 1). On the fourth day of indomethacin treatment, her fever of 39 °C recurred and was associated with increased weakness, malaise, and confusion. That night, two episodes of right hemiparesis with aphasia of about 30 min duration were observed. No areas of ischemia or hemorrhage were seen by magnetic resonance imaging (MRI)-angiography of her brain. An analysis of her cerebrospinal fluid found 282 mg/dL of protein, but there was no increase in cell number (Table 3). At this point, her symptoms were treated with dexamethasone 16 mg daily for 2 days followed by methylprednisolone 1 g daily for the next 4 days and then again dexamethasone 16 mg daily. Ceftriaxone was substituted by meropenem, and empiric anti-TB treatment and treatment with fluconazole were added. Corticosteroid therapy was followed by an impressive clinical improvement, including defervescence, regaining orientation, and regaining her ability to stand and to eat. However, on the fourth day of corticosteroid treatment, her high fever recurred together with extreme weakness, anorexia, and episodes of confusion.

**Table 3:** Laboratory values

Analyte	Reference	Day 1	Day 12	Day 22	Day 29
Leukocyte count (×10^3^/μL)	4.3–11.0	**2.9**	**1.6**	4.8	5.9
Polymorphonucleocytes (×10^3^/μL)	1.9–8.0	**1.3**	**1.0**	3.7	5.1
Lymphocytes (×10^3^/μL)	1.0–3.7	1.0	**0.3**	**0.5**	**0.3**
Hemoglobin (g/dL)	12–16	**9.7**	**8.2**	**10.9**	**9.3**
Platelet count (×10^3^/μL)	140–450	151	**77**	**68**	**58**
Triglycerides (mg/dL)	30–150	128	**296**	**301**	**442**
Fibrinogen (mg/dL)	180–500	-	474	**94**	**98**
Ferritin (ng/mL)	13–150	**309**	**673**	**1046**	**733**
sIL-2R(CD25) (kU/L)	223–718	-	**13,190**	**15,730**	**45,800**
sIL-2R/ferritin ratio	<2*	-	**20**	**15**	**62**
Lactate dehydrogenase (U/L)	120–230	**265**	**412**	**451**	**454**
Aspartate transaminase (IU/L)	<40	13	23	**76**	**59**
Alanine transaminase (IU/L)	<40	12	17	**59**	**68**
Total bilirubin (mg/dL)	<1.4	0.4	0.3	0.5	**2.8**
Conjugated bilirubin (mg/dL)	<0.3	0.2	0.2	**0.4**	**2.6**
Albumin (g/dL)	3.5–5.2	**2.8**	**2.8**	**2.1**	**1.7**
Erythrocyte sedimentation rate (mm/h)	<30	**81**	**65**	-	-
C-reactive protein (mg/dL)	<0.50	**0.62**	**9.9**	**16.8**	**8.2**
Lupus anticoagulant panel					
aPTT-low phospholipid	<1.15		0.85		
DRVVT ratio	<1.10		**1.3**		
Cerebrospinal fluid					
Cells (/μL)	≤5		0		4
Glucose (mg/dL)	50–75		60		**39**
Protein (mg/dL)	15–45		**282**		**152**
Chloride (mmol/L)	120–132		**113**		**107**
Lactate (mmol/L)	1.1–2.2		**2.9**		**6.6**
Link index	<0.7		0.5		0.5
Widal Wright reaction to					
Salmonella O antigen	≤1:160		1:160		1:160
Salmonella H antigen	≤1:160		**1:2560**		**1:2560**

High-resolution human leukocyte antigen (HLA) typing: HLA-A *32:01, HLA-B *40:02, *51:01, HLA-C *02:02,16:02, HLA-DRB1 *11:01,*11:04, HLA-DQB1 *03:01

Negative results: repeated blood cultures; QuantiFERON-TB test, search in blood for plasmodia, *Plasmodium* antigen, *Leishmania* immunoglobulin G (immunoblotting), hepatitis C virus antibody, *Legionella pneumophila* antibody, *Mycoplasma pneumoniae* antibody, *Coxiella burnetii* antibody, *Bartonella henselae* antibody, *Brucella* antibody, *Borrelia burgdorferi* antibody, *Treponema pallidum* antibody, HIV antibody, hepatitis B virus DNA, cytomegalovirus DNA, Epstein-Barr virus DNA, human herpesvirus6 DNA, parvovirus immunoglobulin M; urine cultures; search in urine for *Legionella* antigen; search in bone marrow aspirate for *Leishmania*, *M. tuberculosis* DNA; antinuclear antibodies

Values out of the reference range are in **bold**; normal routine laboratory values are not listed

^*^A ratio >2 is observed in most patients with lymphoma-associated hemophagocytic lymphohistiocytosis [50, 51]

*aPPT* activate partial thromboplastin time, *DRVVT* dilute Russell’s viper venom time, *sIL-2R* soluble interleukin-2 receptor

The criteria for HLH/MAS were fulfilled (Table 1), but no underlying cause was evident. Because eight cases of AOSD associated with SBIs have been reported (see below), the silicone breast prosthesis of our patient was considered to have possibly contributed to her hyperinflammatory state, and consequently was explanted on the 22nd hospital day. A more detailed medical history revealed that the breast prosthesis had caused our patient many problems. Because of painful inflammation, the first prosthesis had to be explanted after 6 weeks and necrotic inflammatory tissue surrounding the prosthesis removed. Because an infection was suspected, the prosthesis was cleaned with antiseptics and then re-implanted, but no infectious agent could be isolated. Two months later, the prosthesis had to be removed definitively. Again, necrotic inflammatory tissue had to be removed and no infectious agent could be isolated. One year prior to her current hospitalization, a new silicone breast prosthesis was implanted. An inflammatory reaction with swelling of her breast was treated locally with infiltration of corticosteroids. Thereafter, our patient could tolerate the prosthesis and a mild pain was felt only when touching the prosthesis.

**Fig. 2:**
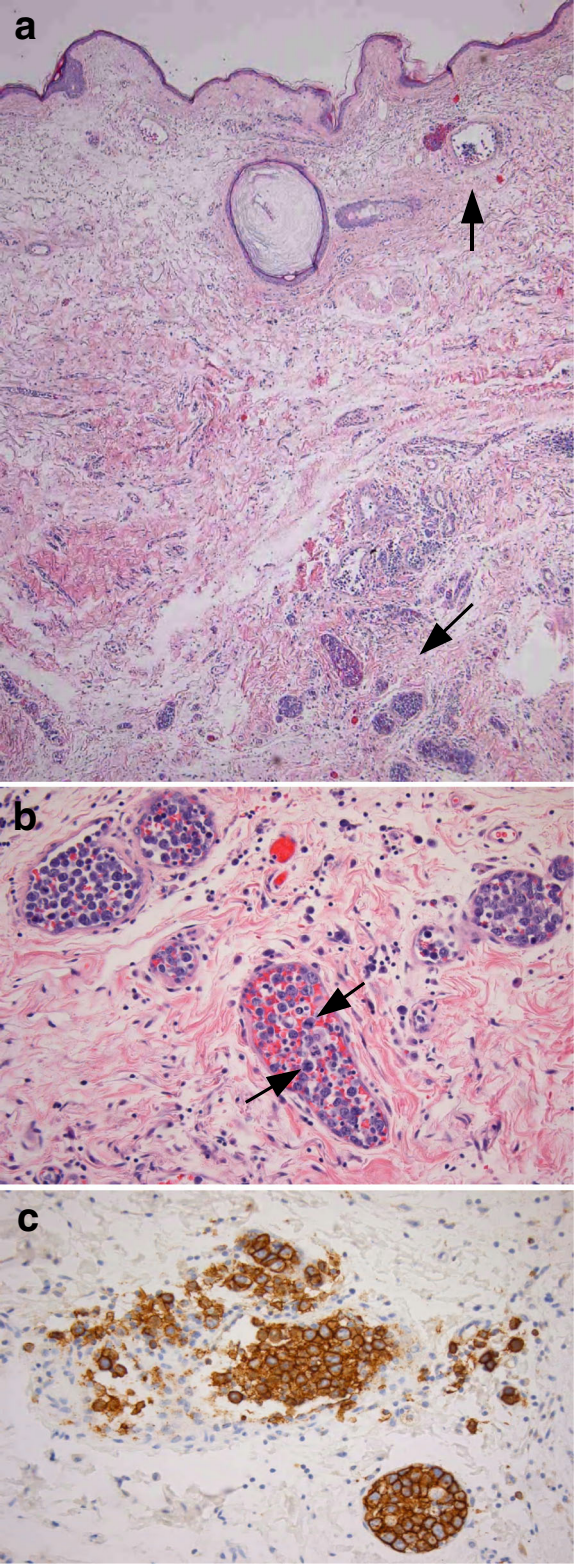
**a** Skin with enlarged vessels filled with tumor cells (*arrows*) mainly in the deep dermis (hematoxylin eosin stain, low magnification). **b** Intermediate vessels with tumor cells and some mitotic figure (*arrows*). **c** The tumor cells are strongly positive for CD20 by immunohistochemical reaction for CD20

Significant worsening occurred after removal of the intact prosthesis. Her fever rose up to 40.4 °C, she became icteric (Table 3) and for the first time she experienced severe low back pain that radiated to her lower legs and required analgesic treatment with opium derivates. She complained of perianal anesthesia and fecal incontinence. The day of implant removal, 25 g of endovenous immunoglobulin was started and given daily for 3 days to treat the MAS. Finally, cyclosporine 100 mg twice daily was administered without any benefit. On the 29th hospital day, the results of the histologic examination of the tissue taken at implant removal were available and showed (a) normal pectoralis muscle with absence of neoplastic infiltration; (b) cutis and subcutis with dilated vessels showing an intraluminal proliferation of atypical large lymphocytes with a vesiculosus nucleus with several nucleoli (Fig. 2). A immunohistochemical analysis of the neoplastic cells revealed positivity for cluster of differentiation (CD) 20, B-cell lymphoma (BCL)-6, BCL-2, multiple myeloma oncogene 1 (MUM-1), and weaker staining for CD5. The proliferation index Ki-67 was over 90 %. Results for terminal deoxynucleotidyl transferase (TdT), CD10, CD34, CD3, and cytokeratin-CAM were negative. These results are consistent with the diagnosis of IVLBCL. The remaining parenchyma showed an extensive giant cell-lymphohistiocytic foreign body reaction, but no carcinomatous infiltration. Her familial medical history was notable for an uncle who has died from acute lymphatic leukemia at 43 years of age.

Our patient was transferred to our Hematology Ward where she was treated with six cycles of R-CHOP [rituximab, cyclophosphamide, hydroxydaunorubicin (doxorubicin), vincristine, prednisone] chemotherapy. Intrathecal prophylaxis with methotrexate was also given. After 6 months her white blood cell count, platelet count, hemoglobin level, LDH level, and liver function tests were again within the reference ranges. After 2 years our patient’s clinical status is good, but sequelae of the cauda equina syndrome are still present, including low back pain, saddle anesthesia, difficulties in voiding the bladder and intestine, loss of sexual sensation, right plantar numbness, and gait instability. She continues to undergo 3-monthly follow-up visits (see Additional file 1 for supporting data).

## Discussion

### 1. Diagnostic considerations

Elevated soluble interleukin-2 receptor (sIL-2R/CD25) has been observed in sera of patients with malignant lymphoma, and can therefore be used as a diagnostic and prognostic marker for malignant lymphoma [49]. sIL-2R was shown to be released from activated T cells mainly due to cleavage by matrix metalloprotease-9 (MMP-9) produced by tumor-associated macrophages [49]. Elevated sIL-2R is also seen in HLH and has been proposed as a diagnostic marker of the disease (Table 1). Secondary HLH can be caused by malignant lymphoma too [1–4]. HLH is considered characteristic of the so-called Asian variant of IVLBCL [38, 39]. A sIL-2R/ferritin ratio >2 has been observed in most patients with malignant lymphoma-associated HLH and only rarely in those with benign disease-associated HLH. Therefore, a high sIL-2R/ferritin ratio has been proposed as a useful marker for the diagnosis of a lymphoma-associated HLH [50, 51]. Our patient had sIL-2R/ferritin ratios of 20,15, and 62 (Table 3). Her serum had to be sent for sIL-2R testing to the Reference Center, Dipartimento Interaziendale e Medicina di Laboratorio, University of Padua, Italy, and the results were available 3 weeks later, when the histologic diagnosis of IVLBCL was already known. In order to be of diagnostic utility, sIL-2R testing results have to be available in a shorter period of time.

Autoptic or intra vitam diagnosis of IVLBCL of the adrenal glands in patients manifesting adrenal insufficiency has been reported [52–54]. Primary or secondary adrenal gland infiltration was detected in up to 67 % of patients diagnosed with IVLBCL [53]. Because the adrenal gland has a sufficient reserve, development of adrenal insufficiency requires destruction of 90 % of the adrenal gland tissue [53]. Indeed, a silent IVLBCL initially manifesting as a unilateral adrenal incidentaloma has been described [55]. In this case an abdominal CT scan performed on a regular follow-up appointment revealed asymptomatic bilateral enlargement of the adrenal glands, while the adrenal function remained normal, suggesting a relatively slow progress of the disease. An ^18^FDG-PET/CT showed an exclusive, strong uptake in both adrenal glands that was absent at a control one year after R-CHOP chemotherapy [55]. In our patient, the diagnostic significance of the exclusive hypermetabolic adrenals demonstrated by ^18^FDG-PET/CT carried out 7 weeks before the histologic diagnosis of IVLBCL was not recognized. Only adrenal tuberculosis was considered. Serum ACTH and cortisol levels were within the normal range. At that time, a biopsy of her adrenal glands [55], a random transbronchial biopsy of her lungs [47, 48], a random biopsy of her skin [46], or a biopsy of her liver probably could have facilitated an earlier diagnosis.

### 2. HLA-DRB1*11, HLA-DQB1*03, and siliconosis

Foreign body reaction to a silicone implant and capsule formation is a physiological process that protects the organism from a non-degradable foreign material that is too large to be engulfed by macrophages [11, 36]. However, this physiological process is highly variable and can lead to local inflammatory symptoms, an excessive capsular fibrosis, contracture in 2–50 % of patients, and sometimes systemic symptoms and disease [10–36]. Excessive capsular fibrosis and capsular contracture are well-recognized adverse events. However, harmful systemic effects of silicone, most notable connective tissue diseases (CTDs) or systemic autoimmune phenomena, remain controversial [13, 34]. A meta-analysis rejected the presumed relationship between SBIs and CTD [13, 34] but, in seeming contradiction, case reports about CTDs and systemic symptoms continue to be published [10, 23, 24]. As for other rare diseases or side effects [56], most epidemiological studies lack enough power to show significant associations [13]. In addition, most studies determined the risk of development of CTDs shortly after breast implantation, which may result in false-negative results, given that most patients complain only years after the breast implant operation as reported in other studies [10]. Another shortcoming of most epidemiological studies is that they focus on classic CTDs, ignoring other (systemic) autoimmune phenomena recently classed as ASIA syndrome [9–11, 13].

It seems obvious that a favorable genetic or predisposing background plays a key role in the development of rare diseases or side effects. Silicone gel was shown to enhance the development of autoimmune diseases in New Zealand black mice but failed to induce it in BALB7cAnPt mice [57]. Thus, it was hypothesized that silicone could play a similar role in the development of autoimmune diseases in a small percentage of women who are genetically susceptible to such diseases [57]. More than 200 genetic loci have been shown to be associated with one or more autoimmune disorders [58]. It has been suggested that most of these genetic associations reflect the immunoregulatory effect of the HLA molecules themselves [58]. For many of these disorders, genes within the major histocompatibility complex (MHC) have by far the strongest single genetic effect [58]. The human MHC is the most gene-dense region of the human genome and encodes the most polymorphic human proteins [59]. Its variants are associated with more than 100 different diseases, mostly autoimmune, infectious, or inflammatory diseases [60]. However, identification of causal variants within the MHC for the majority of these diseases has remained difficult due to the great variability and extensive linkage disequilibrium that exist among alleles throughout this locus [61]. In one study, 77 symptomatic patients with SBIs were reported to share important genetic characteristics (primarily HLA-DR53 = HLA-DRB4) that differentiate them from their asymptomatic controls [62]. Another study showed that all nine women with post-silicone implant systemic sclerosis had HLA-DQ5 or DQ7 (HLA-DQB1*0301) alleles [63]. Compared to 128 healthy Caucasian controls, these nine women had a significantly lower frequency of the hydrophobic leucine at position 26 of the first domain of the HLA-DQB1 allele [63]. Eleven women who had developed inflammatory myositis after they received silicone implants differed by increased frequency of HLA-DQA1*0102 from 76 women with myositis without silicone implants [64]. Two sisters who received SBIs developed polyarthritis and neurological symptoms but experienced dramatic improvement after implant removal. HLA typing revealed HLA-DRB1*0405, HLA-DQB1*0302, and HLA-DRB4*01 [21]. Three sisters who carried the BRCA-1 gene mutation had a preventive mastectomy and were reconstructed with SBIs. After the reconstruction, all three patients developed fatigue, arthralgia, myalgia, and sleep disturbances within a period of 4 years. Silicone implant replacement by non-silicone gel containing Monobloc Hydrogel breast implants was followed by improvement of all complaints in the three sisters [23]. These reports further indicate that the susceptibility to develop silicone implant incompatibility syndrome or ASIA may be genetically determined. HLA typing of all who receive silicone implants would enable physicians to better define the risk of adverse side effects of silicone implants.

### 3. Siliconosis and macrophage activation syndrome

In the animal model, silicone (dimethylpolysiloxane) exhibited potent adjuvant properties that varied according to its molecular weight and chemical composition [57, 65–68]. Silicone elastomer, pre-adsorbed with plasma proteins, activates human monocytes/macrophages in vitro to secrete IL-1ß, IL-6, and TNF-α [68]. In female Wistar rats it was shown that silicone induced persistent recruitment of leukocytes at the site of the injection and that macrophage activation was still present 45 days after injection. Activated macrophages exhibited an increased expression of adhesion and co-stimulatory molecules and an enhanced production of oxidant metabolites and nitric oxide (NO) [69]. In a study on biomaterial-induced macrophage activation it was shown that concentrations of markers of monocyte/macrophage activation, the chitinase-like proteins chitotriosidase and YKL-40, were significantly higher in patients with certain bio-implants and that these markers rose progressively as adverse reactions evolved [70]. A macrophage-specific PET imaging probe has recently been developed to image implant-surrounding activated macrophages and, thus, to evaluate in vivo medical device-associated inflammation [71].

Even if a silicone gel-filled breast implant does not rupture, small amounts of low molecular weight fluid dimethylpolysiloxane may permeate (bleed or sweat) out of the implant into the surrounding tissue [69]. Transcapsular migration of silicone particles from the mammary implant through the fibrous capsule of mammary prosthesis has been observed [72]. Silicone was identified within macrophages residing in the synovia and skin of patients with SBIs and CTD [73, 74]. One mechanism that has been proposed to explain an interaction between silicones and the immune system is the following. After silicones have been freed from the implant, they come into contact with native tissue, which may lead to denaturation of tissue macromolecules such as fibronectin, fibrinogen, and apolipoprotein B. The denatured macromolecules are sufficiently different from native tissue to be recognized as foreign, which may trigger activation of the immune system and lead to the generation of antibodies against them. These antibodies may cross react with native tissue, generating an autoimmune response [75].

Eight cases of AOSD associated with SBIs have been reported [76–80]. Improvement of AOSD was described in two of three patients in whom the SBI was removed [76]. AOSD is a systemic inflammatory disorder, a hyperferritinemic or cytokine storm syndrome that has similarities and pathophysiological overlap with MAS/secondary HLH [4, 7, 81]. AOSD has therefore also been defined as an autoimmune-associated, reactive, or secondary HLH [7, 81]. NK cytotoxicity was found to be reduced in AOSD [82]. Even if MAS has not been previously associated with SBIs, considering these studies it seems plausible that in our patient her SBI could have contributed to the development of MAS.

### 4. HLA-DRB1*11, HLA-DQB1*03, and lymphoma

There are a few studies that show a higher frequency of HLA-DRB1*11 and/or HLA-DQB1*03 in patients with hematologic malignancies [83], non-Hodgkin’s lymphoma, diffuse large B-cell lymphoma [84], mycosis fungoides (cutaneous T-cell non-Hodgkin’s lymphoma) [85–88], acute lymphoblastic leukemia [89], chronic lymphocytic leukemia [90], hairy cell leukemia (B-cell leukemia) [91], and HCV virus-associated lymphoma [92] compared to controls. These data indicate a probable genetic basis of the lymphomagenesis. Our patient’s uncle died at 43 years of age from acute lymphoblastic leukemia. The occurrence of more cases of hematologic malignancies in a family further suggest a genetic contribution to the pathogenesis [87, 88].

HLA-DRB1*11 or HLA-DQB1*03 have been associated with, among others, the autoimmune disease systemic sclerosis [93, 94]. Systemic sclerosis, like other autoimmune diseases, has been associated with lymphomas [95, 96]. Even the rarely occurring intravascular large B-cell lymphoma has been described in a woman with systemic sclerosis [97].

### 5. Siliconosis and lymphoma

Silicosis is a known risk factor for lung cancer [98]. Can siliconosis be considered a risk factor for cancer too? Although non-Hodgkin lymphomas of the breast are exceedingly rare, cases of breast implant-associated anaplastic large cell lymphoma (ALCL) continue to be reported [99–101]. The finding of more ALK-1-negative ALCL among breast implant-associated ALCLs compared to ALCL with breast involvement in women without breast prosthesis was considered as evidence in favor of an association between silicone breast prosthesis and ALK-1-negative ALCL [99]. Also, because the breast implant-associated ALK-1-negative ALCL has distinct clinical features, for example, a better prognosis, compared to non-implant-associated ALK-1-negative ALCL, it has been proposed as a new clinical entity [99–102]. The lack of strong epidemiological evidence for a causative role of breast implants most likely reflects the extreme rarity of the disease [102]. If siliconosis could have contributed to the pathogenesis of IVLBCL in our patient, one could speculate whether removal of the SBI and therefore removal of the chronic inflammatory stimulus could have improved disease prognosis.

Inflammation has been linked with tumorigenesis and tumor progression in many malignancies [98]. Chronic inflammation can promote tumor initiation and progression but can also protect against cancer through immune surveillance [103]. Chronic inflammation can increase the risk of cancer by providing bioactive molecules including cytokines, chemokines, and growth factors. These bioactive molecules maintain a sustained proliferation rate, interfere with apoptosis, promote angiogenesis, and induce the production of free radicals and reactive oxygen and nitrogen species (RONS) [98]. TNF-α, the prototypical proinflammatory cytokine, can exert pro-apoptotic and anti-apoptotic effects [98]. IL-6, another major mediator of inflammation, serves to block apoptosis in cells during the inflammatory process, keeping them alive, protecting them from cellular apoptotic deletion, and allowing them to progress towards neoplastic growth [104]. In cancers associated with chronic inflammation, either excessive free radical production or defective antioxidant mechanisms or both of these were observed [98]. Free radicals, RONS, can instigate carcinogenesis either directly by reacting with and damaging DNA and RNA or indirectly by interfering with transcription factors, for example, NF-kB [98, 105]. Free radicals and cytokines can both either induce or become induced by NF-kB [103]. As a consequence, a positive feedback loop exists between the ability of inflammatory cytokines to induce the synthesis of RONS and of RONS to elicit the generation of inflammatory cytokines [103].

Lymphoid neogenesis and lymphoma are associated with chronic inflammation in infections like chronic hepatitis C or *Helicobacter pylori* infection, and in chronic autoimmune diseases [98, 106]. Considering the key role of B- and T-cell activation in the pathogenesis of infections, autoimmune diseases, and lymphoma, it is perhaps not surprising that longstanding chronic inflammation and/or antigen stimulation have emerged as major predisposing factors of lymphoma in patients with these diseases [106].

### 6. Familial hemophagocytic lymphohistiocytosis- related gene mutations and macrophage activation syndrome

The complete cell-mediated cytotoxicity defect due to bi-allelic disruptive mutations in one of the familial HLH-related genes leads to full-blown familial HLH early in life, with a peak incidence between 1 and 6 months of age and with the typical and rapidly fatal course. However, the clinical impact of less complete or partial defects in this pathway remains to be clarified [2]. Mutations that lead to partial insufficiency of the cytotoxic machinery (missense mutations, single nucleotide polymorphisms, monoallelic mutations) have been found in patients with later-onset familial HLH [2, 107], in patients with secondary HLH including MAS [6, 108–111], and in those with other diseases, such as systemic juvenile idiopathic arthritis [112, 113] or lymphoma (see below). Therefore, distinctions between familial and secondary HLH are becoming increasingly blurred as new genetic causes are identified [6].

### 7. Familial hemophagocytic lymphohistiocytosis-related gene mutations and lymphoma

Mutations of the *perforin* gene and the *UNC13D* gene, both familial HLH-related genes, have been found in 27 % of children with ALCL [114, 115]. These data suggest that impaired cytotoxic machinery may represent a predisposing factor for ALCL. In one study, 11 out of 23 patients with bi-allelic *perforin* gene mutations, whose onset of familial HLH was delayed or abrogated, presented with B- or T-cell lymphoma or acute or chronic leukemia as the primary clinical illness [116]. In the mouse model of lymphomagenesis, *perforin* was shown to act as a suppressor of B-cell malignancies [117].

NK cells (innate immunity) and CTLs (adaptive immunity) are recognized for their role in the defense against viruses, in immune surveillance against a variety of malignancies, and in downregulating or ending an immune response [118–121]. These roles are performed by perforin initiating the apoptosis of dangerous cells, either those harboring an intracellular pathogen, those possessing the potential for uninhibited growth and spread, or those antigen-presenting cells and activated T cells that have just accomplished their immune response function. Perforin is a pore-forming protein stored in secretory granules of CTLs and NK cells that synergizes with the pro-apoptotic serine proteases (granzymes) to deliver a lethal hit to the target cell [118–121]. It is possible that in our patient polymorphisms of the familial HLH-related genes, the genes encoding the cytotoxic machinery, had a causal role for both the MAS and the IVLBCL. Unfortunately, we have not had the chance to sequence these genes.

## Conclusions

**Fig. 3:**
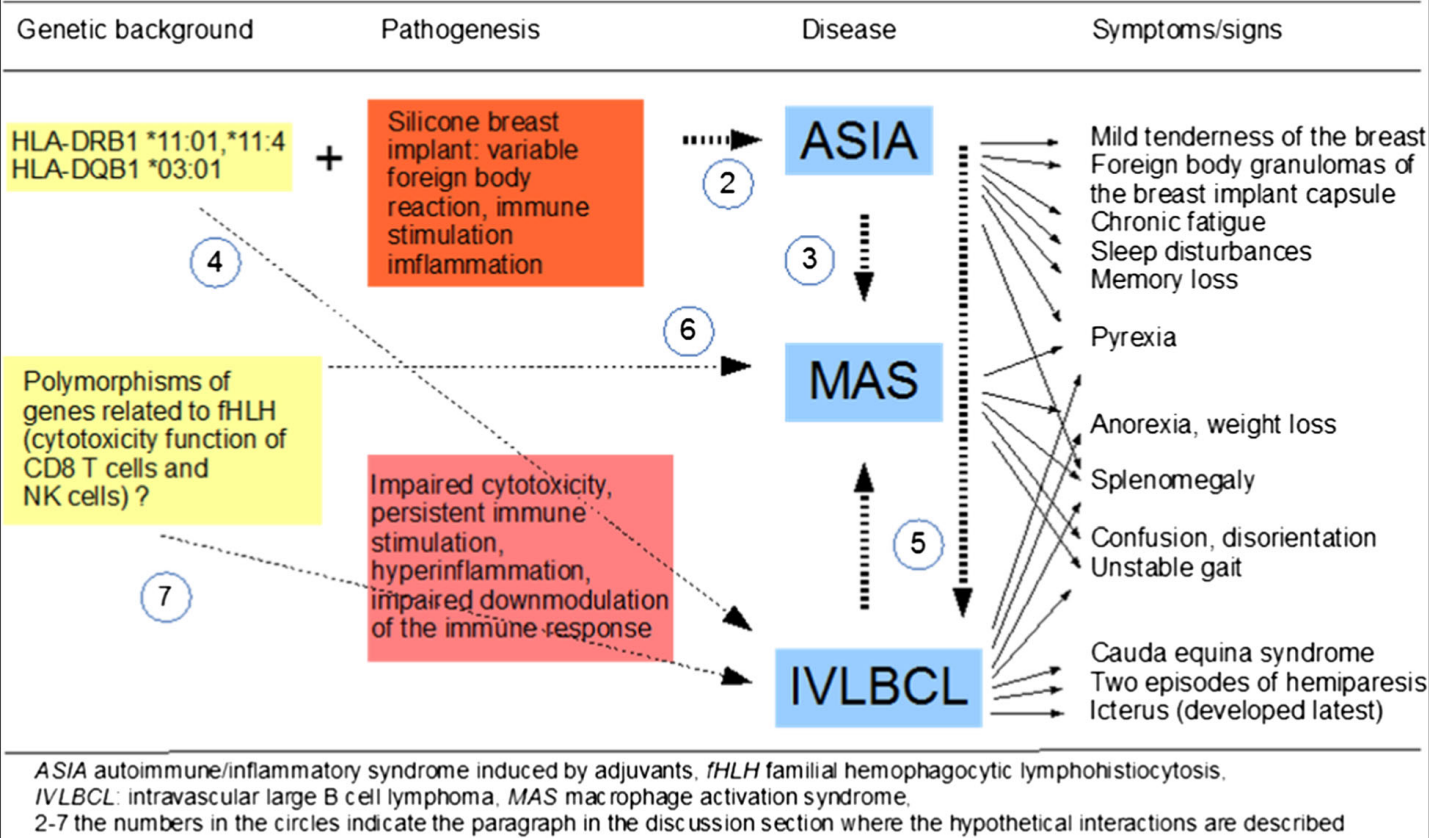
Hypothetical interactions between the different syndromes and diseases of the described patient

To the best of our knowledge, this is the first reported case of IVLBCL associated with an SBI. MAS, siliconosis/ASIA, and IVLBCL are rare diseases and are difficult to diagnose. The combination of these three rare conditions suggests multifactorial interactions (Fig. 3). When IVLBCL is considered in the differential diagnosis of fever of unknown origin, then a diagnosis can be made by random skin biopsy or by biopsy of a suspected organ indicated by ^18^FDG-PET/CT. The chronic inflammation of siliconosis in our patient might have contributed to the development of her IVLBCL. MAS/secondary HLH is a known manifestation of IVLBCL. Siliconosis might also have predisposed our patient to MAS development, as there are case reports of siliconosis associated with AOSD, another cytokine storm syndrome like MAS. Hypothetical mutations of genes causing familial HLH could have favored both MAS and lymphoma development. Future expansion of genetic testing will further clarify the role of polymorphisms of genes encoding the cytotoxic machinery in the pathogenesis of hematologic malignancies, and possibly will discover polymorphisms of further genes involved in the pathogenesis of lymphoma, secondary HLH, and autoimmune diseases. HLA testing of all women who undergo silicon breast implantation could enable physicians to evaluate the risk of getting siliconosis/ASIA, a still-debated disease.

## Abbreviations

^18^FDG-PET/CT, ^18^Fluorodeoxy-glucose positron emission tomography/computed tomography; ALCL, anaplastic large cell lymphoma; ALK-1, anaplastic lymphoma kinase-1; AOSD, adult-onset Still’s disease; ASIA, autoimmune inflammatory syndrome induced by adjuvants; BCL, B-cell lymphoma; CD, cluster of differentiation; CT, computer tomography; CTD, connective tissue diseases; CTL, cytotoxic T cell; HLH, hemophagocytic lymphohistiocytosis; IVLBCL, intravascular large B-cell lymphoma; LDH, lactate dehydrogenase; MAS, macrophage activation syndrome; MHC, major histocompatibility complex; MMP-9, matrix metalloprotease-9; MRI, magnetic resonance imaging; MUM-1, multiple myeloma oncogene 1; NK, natural killer; RA, rheumatoid arthritis; R-CHOP, rituximab, cyclophosphamide, hydroxydaunorubicin (doxorubicin), vincristine, prednisolone; RONS, reactive oxygen and nitrogen species; SBI, silicone breast implant; sJIA, systemic juvenile idiopathic arthritis; SLE, systemic lupus erythematosus; TB, tuberculosis; TdT, terminal deoxynucleotidyl transferase; TNF-α, tumor necrosis factor alpha

## Additional file

Additional file 1: Availability of data and supporting materials. (DOCX 18.6 kb)
