# Supplementary material for: Intravascular large B-cell lymphoma associated with silicone breast implant, HLA-DRB1*11:01, and HLA-DQB1*03:01 manifesting as macrophage activation syndrome and with severe neurological symptoms: a case report
Source: J Med Case Rep. 2016 Sep 15;10:254. doi: 10.1186/s13256-016-0993-5 (PMC5025582; doi:10.1186/s13256-016-0993-5)
Supplement: Additional file 1: — Availability of data and supporting materials. (DOCX 18.6 kb) [file 13256_2016_993_MOESM1_ESM.docx]

Supporting materials for the manuscript “Intravascular large B cell lymphoma associated with silicone breast implant, HLA-DRB1*11:01, and HLA-DQB1*03:01 manifesting as macrophage activating syndrome and with severe neurological symptoms: a case report”

| Follow up laboratory data | | | | | | | |
| --- | --- | --- | --- | --- | --- | --- | --- |
| Analyte | Reference | Month  4 | Month  6 | Month  9 | Month  12 | Month  15 | Month  21 |
| Leukocyte count (x10^3^/µL) | 4.3-11.0 | **2.6** | 4.4 | 4.6 | 4.0 | 4.0 | 5.2 |
| Polymorphonucleocytes (x10^3^/µL) | 1.9-8.0 | **1.8** |  | 1.93 | **1.7** | **1.7** | 2.1 |
| Hemoglobin (g/dL) | 12-16 | **10,8** | 13.7 | 14.0 | 13.3 | 13.3 | 13.9 |
| Platelet count (x10^3^/μL) | 140-450 | 180 | 181 | 180 | 180 | 175 | 196 |
| Ferritin (ng/ml) | 13-150 |  |  |  | 94 | 76 |  |
| LDH (U/L) | 120-230 |  |  | 164 | 172 | 153 | 162 |
| AST (IU/liter) | <40 | 27 | 30 |  |  |  | 34 |
| ALT (IU/liter) | <40 | **73** | 33 |  |  |  | 27 |
| Total bilirubin (mg/dL) | <1.4 | 0.5 |  |  |  |  |  |
| Conjugated bilirubin (mg/dL) | <0,3 | 0.2 |  |  |  |  |  |
| Creatinine (mg/dL) | 0.50-1 |  |  | 0.7 | 0.7 |  |  |
| Cerebrospinal fluid |  |  | Normal values |  |  |  |  |

**Cerebral MRI (month 4):** periventricular leukoencephalopathy (toxicity of chemotherapy?)

**^18^FDG-PET/CT (month 4):** no abnormalities

**CT of the dead, thorax and abdomen (month 6):** no intracranial abnormalities; lungs with biapical scars; pericardial fluid 4 mm in diameter; no enlarged mediastinal lymph nodes; liver enlarged, spleen bipolar diameter of 12 cm; no enlarged intraabdominal or retroperitoneal lymph nodes; spondylosis on the lumbar spine.

**MRI of the thoracolumbar spine (month 6):** disc protrusions T5-T6, L3-L4, L4-L5, L5-S1; no enhancement following contrast medium administration.

Answers to reviewer comments

*Case management 
Reasons for not following the HLH 2004 protocol  
French study protocol - In this protocol, patients receive prednisolone, anti-thymocyte globulin (ATG), and Cyclosporine A, which is followed rapidly by HSCT which offers comparable survival to etoposide based regimens. The standard of care remains the etoposide/dexamethasone immune-chemotherapy until further results are obtained. (1)
Reason for choice of steroid - Regimen consisting of dexamethasone with prednisolone was used*

The HLH 2004 protocol provided treatment recommendations for both familial HLH and reactive HLH including MAS that utilize corticosteroids, cyclosporine, and etoposide as a bridge to transplant. However, it is increasingly recognized that most disorders meeting HLH 2004 criteria will not require such aggressive immunosuppression. Additionally, chemotherapeutic strategies similar to the HLH 2004 protocol carry a high risk for iatrogenic infection and hematologic malignancy. Therefore, the management of MAS is far from standardized and remain controversial. Most agree that high doses of corticosteroids are useful. While many practitioners treat MAS with HLH-2004-like approach, less toxic strategies that include cyclosporin, endovenous immunoglobulin, IL-1- or IL-6 blockers have emerged (References 1,4,8).

Most colleagues involved in the treatment of the reported patient were not convinced of the diagnosis of MAS and therefore hesitate to prescribe etoposide. High dose corticosteroids were followed by prompt significant improvement, but this improvement lasted only a few days. Because the addition of endovenous immunoglobuline and cyclosporine seemed ineffective the breast silicone implant was removed with the intention to remove a possible trigger of MAS. Unexpectedly the histologic examination of tissue adjacent to the prosthesis revealed an ILBCL.

We didn't wont to discuss therapy because we won't burst the frame of an already complex clinical case. Therapy is best dealt with in many review articles including those cited in this case report. The uniqueness of this case report is the combination of the three rare syndromes with their diagnostic and pathogenetic implications.

*Radiological confirmation of Spinal involvement.*

*A Demyelinating process seems to underlie the neurological manifestations although no attempt has been made to confirm the same by way of radiological or electroneurophysiological studies.
This needs to be explained as the patient has completed one and a half years of follow up successfully.*

RMI imaging showed merely disc protrusions that did not explain the cauda equina syndrome

*Use of Naranjos algorithm or the WHO UMC assessment to suggest causality.*

Causality is suggested by the analogy of the silicone implant associated ALK negative anaplastic large cell lymphoma (Discussion subheading 5) and by localization at the side of chronic inflammation (Discussion subheading 5). It seems obvious that for very rare diseases a genetic basis might play a role (Discussion subheadings 2,4,6,7) but collection of further data e. g. HLA determinations or search for polymorphisms of genes related to fHLH are necessary in order to advance this knowledge directed towards a personalized medicine.

*Serum Ig G levels to be documented as rising titre of the same could provide necessary evidence of the causative association between Silicone implants and autoimmunity.(2)*

Serum IgG levels were repeatedly within the reference range. Fulfilled major criteria for ASIA are: i) exposure to an external stimulus (silicone), ii) “typical” clinical manifestations (sleep disturbances since silicone prosthesis implantation) the other symptoms cannot be differentiated from those of IVLBCL, iii) extensive giant cell-lymphohistiocytic foreign body reaction of the tissue adjacent to the silicon implant.

*Significance of later development of Icterus in figure needs to be explained further as the same has been excluded from the text.*

The icterus can be seen in relation to the “cytokine storm” and potential multiorgan involvement of MAS (Reference 7) and /or the potential multiorgan involvement of IVLBCL (References 37-40). Additional inflammation due the surgical removal of the silicone prosthesis or side effects of the general anesthesia might have been further contributing factors to the increased bilirubin level.
 *Introduction 
The focus is squarely on the role of silicone implants in the pathogenesis of the condition.*

Indeed, the association between silicone implant and intravascular large B cell lymphoma (ILBCL) is unique and has not been reported previously. In the cited literature there are hints suggesting a possible/probable causative association between the chronic inflammation due to the silicone implant (ASIA) and the IVLBCL.

*The title suggests the role of HLA polymorphism in the condition which seems to have been overlooked in the introduction.*

The combination of the three rare and difficult to diagnose syndromes, autoimmune/inflammatory syndrome induced by adjuvant (ASIA), macrophage activation syndrome (MAS), and ILBCL is unique and has not been reported previously. In the cited literature there are indications of interactions between these three rare syndromes, represented in Fig. 3. Because these three rare syndromes may not be well known to the common reader they are introduced in the introduction section. The role of HLA polymorphisms is extensively treated in the discussion section (paragraph 2,4)..
 *Clinical features, Investigations and management need to be incorporated into a flow diagram or a time line highlighting the exact chronological sequence of events.*

A time line of the main clinical features has been constructed as Fig. 1.

*Clinical significance of point 6 in discussion needs to be elaborated further.*

Polymorphisms of genes related to fHLH may underlay also MAS (Reference 2,6,107,112,113). Its clinical significance might be elaborated further in the future when more research data will be available.

*The recommendation for routine HLA testing for every individual who is supposed to undergo a breast augmentation/mammoplasty seems overreaching and without requisite clinical evidence at present.*

The routine HLA testing for every individual who is supposed to undergo a silicon prosthesis implantation is recommended in order to collect further data, that may allow in future to better evaluate the risk of potential important side effects.

*Factual accuracy
The note below the table depicting the diagnostic criteria for ASIA syndrome states that the criteria were taken from 93 cases who were vaccinated with the Hepatitis C Vaccine, whereas the original criteria were proposed by Dr Yehuda Schoenfeld*

…….Table 2 Suggested criteria for the diagnosis of ASIA^9^ …….Reference 9 is the article where Dr. Yehuda Shoenfeld coined the definition of ASIA. Reference 12 cited below the table, reported the evaluation of the proposed criteria in 93 cases of ASIA. The article is written by Dr. Yehuda Shoenfeld and his colleagues.
